# An economic analysis of postoperative cataract consultation using remote self-assessment

**DOI:** 10.1371/journal.pdig.0001617

**Published:** 2026-08-14

**Authors:** Casper van der Zee, Janneau L. J. Claessens, Rudy M. M. A. Nuijts, Rob Wouters, Nicolaas J. Reus, Karl Boden, Oliver Findl, Saskia M. Imhof, Robert P. L. Wisse, Miriam P. van der Meulen

**Affiliations:** 1 Ophthalmology Department, University Medical Center Utrecht, Utrecht, the Netherlands; 2 Department of Epidemiology and Health Economics, Julius Center, University Medical Center Utrecht, Utrecht, the Netherlands; 3 Ophthalmology Department, Maastricht University Medical Center+, Maastricht, the Netherlands; 4 Ophthalmology Department, Oogcentrum Noordholland, Heerhugowaard, the Netherlands; 5 Ophthalmology Department, Amphia Hospital, Breda, the Netherlands; 6 Ophthalmology Department, Eye Clinic Sulzbach, Knappschaft Hospitals Saar, Sulzbach/Saar, Germany; 7 Ophthalmology Department, Vienna Institute for Research in Ocular Surgery (VIROS), Hanusch Hospital, Vienna, Austria; 8 Ophthalmology Department, Visibly Inc., Chicago, Illinois, United States of America; Maastricht University Faculty of Health, Medicine and Life Sciences: Maastricht Universitair Medisch Centrum+, NETHERLANDS, KINGDOM OF THE

## Abstract

To evaluate the potential savings realized by replacing the 4–6 weeks consultation following cataract surgery with remote self-assessment. In addition, we evaluated the costs associated with adverse events (AEs) from a societal perspective and evaluated the current cost of AEs based on published literature and publicly available datasets. We determined the cost reduction when replacing the in-person 4–6-week postoperative consultation visit with remote self-assessment using our previous clinical trial, publicly available healthcare resource databases, and threshold analysis to determine the effect on cost if the incidence of AEs is affected by conducting an online self-assessment. We collected AE probabilities and costs of AEs based on published literature and databases. and performed a threshold analysis to demonstrate the financial impact if the incidence of AEs is altered versus the savings due to replacing the 4–6 weeks in-person consultation with remote self-assessment. Remote self-assessments resulted in an average savings between -€83 and -€92 per patient. Physical consultations were less costly only if >80% of remote self-assessments warranted an in-person follow-up. The mean cost of AEs per patient was estimated to be €16.35 and were attributed primarily to rare but severe complications, including endophthalmitis and retinal detachment. Having patients participate in a remote self-assessment following cataract surgery is expected to reduce costs under a wide range of assumptions. Due to the lack of published data regarding the utility loss due to AEs, quality of life was not included in our analysis; nevertheless, these cost savings should be balanced by the clinical impact of potentially missing relatively rare AEs. These findings contribute to the ongoing discussion regarding appropriate care following cataract surgery.

## Author summary

Cataract surgery is the most commonly performed surgical procedure worldwide. After surgery, patients typically visit an eye clinic to detect complications. As the demand for eye care continues to grow, we investigated whether this routine visit could be safely replaced by a remote, self-administered eye test that patients complete at home using a computer or smartphone. We used data from an international clinical trial in which patients tested their own vision online after cataract surgery, combined with national healthcare databases and published literature, to estimate the financial impact of replacing in-person follow-up with remote self-assessment. We calculated the costs of potential complications that might be missed by moving care online, and compared these to the savings from avoiding a physical clinic visit. We found that replacing the routine postoperative visit with remote self-assessment led to cost savings of approximately €83 to €92 per patient across a wide range of assumptions. The average cost of complications per patient was only around €16 — far lower than the roughly €120 cost of a routine in-person consultation. Cost savings were maintained even if remote monitoring tools were imperfect or if societal costs were underestimated. Our findings support a hybrid approach to postoperative cataract care, in which most patients could be monitored remotely, while in-person visits would be reserved for those at higher risk of complications.

## Introduction

Cataract surgery is the most commonly performed procedure; each year, ~ 4.5 million procedures are performed in Europe alone, with low complication rates [1, 2]. As the demand for eye care professionals continues to rise, combined with increasing costs, new solutions are needed in order to increase the accessibility and efficiency [3]. The European Society of Cataract and Refractive Surgeons (ESCRS) recommends that patients receive a postoperative consultation visit 4–6 weeks after surgery in order to measure objective parameters such as visual acuity (VA) and refractive error (RE), which are essential both for quality control and to detect postoperative adverse events (AEs) [4]. However, the ESCRS also states that self-assessment can possibly replace physical, in-person follow-up visits, thereby improving the allocation of resources, decreasing pressure on personnel, and increasing efficiency [5]. Given that currently 98% of follow-up visits in the Netherlands are performed in-person [6], introducing the option for patients to self-assess remotely could be a promising avenue for reducing workload.

We recently published the results of our international prospective Cataract Online Refraction Evaluation Randomized Controlled Trial (CORE-RCT), which investigated the benefits of using remote web-based postoperative self-assessments [7,8]. Because this trial was designed as a clinical validation study, all participants also underwent a comprehensive in-person evaluation 4–6 weeks after surgery for safety and validation purposes, after the self-assessment data were collected. Importantly, this remote self-assessment, which includes both VA and RE, does not require supervision by a healthcare professional. In short, the patients provided valuable information regarding their postoperative outcome remotely using a web-based interface. Web-based VA demonstrated a negligible mean difference compared to the in-person assessment (−0.03 ± 0.14LogMAR). Moreover, the web-based and in-person assessments yielded similar Patient Reported Outcome Measures (PROMs) and AE rates. In contrast, RE was generally overestimated using the online assessment tool, warranting further development.

A critical evaluation of in-person follow-up consultations revealed strong supporters and opponents, thus underscoring the relevance of this research [9–12]. It can be rationalized that the routine 4–6 week visit provides limited marginal value relative to its cost, partly because most vision-threatening complications occur much earlier than the 4–6 weeks postoperative visit (e.g., Visit day 1 or week 1), which are usually still handled in person. Health economics analyses are needed in order to inform the ongoing debate regarding the allocation of limited healthcare resources such as costs and personnel [13]. Here, we evaluated the potential savings associated with replacing the in-person 4–6-week postoperative follow-up visit with a remote self-assessment, assuming non-inferiority in clinical outcome [9–12]. Because the in-person follow-up visits were still performed in the CORE-RCT, non-inferiority could not be tested. Therefore, we also performed a threshold analysis to report on the financial impact if the incidence of AEs was affected due to abstaining from an in-person follow-up ophthalmic exam.

## Methods

### Ethics statement

This study was conducted in accordance with the Declaration of Helsinki, local and national laws regarding research, and European directives with respect to privacy. The CORE-RCT study was registered at ClinicalTrials.gov (NCT04809402) and was approved by the METC (Medical Research Ethics Committee) Utrecht, the Netherlands (NL74625.041.21), the Ethics Committee of the City of Vienna, Austria (EK20-334-0121), and the Ethics Committee of Saarbrücken, Germany (Ha 44/18). Written informed consent was obtained from all participants.

In this health economic analysis, we compared the costs associated with patients performing a remote self-assessment, with the cost of conducting the standard 4–6-week in-person postoperative visit. We first assessed the healthcare resource data obtained in our CORE-RCT. Second, we collected the probability and cost of various AEs from the published literature and existing databases and then multiplied these two values. Third, we performed a threshold analysis to determine the effect on cost if the incidence of AEs is affected by conducting an online self-assessment. The resulting costs were evaluated from an adapted societal perspective, including productivity losses and informal care costs, with a time horizon of three months, unless AE were expected to result in long structural societal burden extending beyond the postoperative period (e.g., visual impairment due to endophthalmitis or retinal detachment)

### Summary of the CORE-RCT

The goal of the CORE-RCT was to determine the accuracy of self-assessed follow-up after cataract surgery.^8^ In short, this tool assesses both VA and RE—including sphere, cylinder, and axis—using a web-based platform developed at Easee BV (currently Visibly Inc.) and then combines this information with self-assessments and Patient Reported Outcome Measures (PROMs), including the Catquest-9SF [14], EQ-5D [15], and NEI-VFQ-25 [16] questionnaires. During the self-assessment the participant uses a smartphone as a remote control to identify optotypes presented on a linked computer screen positioned 3 meters from the participant. The CORE-RCT included 188 eyes scheduled for routine surgery; to be eligible, patients needed to have no ocular comorbidities or predisposing complexities, and they must be both willing and able to perform the self-assessment. Patients were randomized into an unsupervised self-assessment group and a usual care group. Because the RCT was designed as a clinical validation study, after the self-assessment data were collected all participants also underwent a comprehensive in-person postoperative evaluation after 4–6 weeks for safety and validation purposes. The CORE-RCT study revealed that the patients were able to successfully report their postoperative outcome using a web-based platform, although the self-assessment tool overestimated RE, indicating the need for further optimization. Both visual functioning scores—based on the Catquest-9SF and NEI-VFQ-25 results—did not differ significantly between groups. The incidence of AEs was low in both groups, as expected after routine cataract surgery [2]. Since the CORE-RCT was not powered on low incidence rates, this prompted us to extract AEs based on existing national registries. The underlying clinical RCT reported on remote self-assessment vs. physical usual care, and collected healthcare resource data such as scheduled and unscheduled consultations [7, 8]. In the current study, we built upon the results of the RCT and compared the potential cost-saving benefits of undergoing a remote self-assessment and omitting the in-person postoperative visit.

### Healthcare utilization data collected during the RCT

Data regarding the utilization of healthcare resources, transportation, and informal care were collected during the CORE-RCT using a costing questionnaire administered three months after surgery. For transportation, questions were asked regarding the mode of transportation used to travel to the clinic (e.g., by personal vehicle, public transportation, taxi, or other), parking fees where applicable, and distance traveled. Questions regarding informal care asked the patients whether they visited the clinic with support (e.g., a family member, friend, or other caretaker) and if so, whether the support had to miss paid work due to the visit.

### Calculating the current costs associated with postoperative adverse events

Even though AEs are not currently believed to be prevented or treated earlier by conducting routine in-person consultations, the thought of potentially missed AEs could be a bottleneck when implementing exclusively remote self-assessments. Therefore, we estimated the current cost associated with AEs based on published literature sources, expert opinion, publicly available costing data, and the Dutch National Quality Registry for Refractive Surgery. The Principal Investigators of the CORE-RCT were consulted for their combined expert opinion regarding which care or activity is used for a particular AE (e.g., additional visits, new medication, etc.; see ‘*Presumed care consumption*’, Table 1). The nature and incidence of AEs were extracted from the 2024 Dutch National Quality Registry for Refractive Surgery, accessed via https://cataract.dhd.nl. This registry is commensurate with EUREQUO (the European Registry of Quality Outcomes for Cataract and Refractive Surgery) and includes an empirically validated set of postoperative AEs [17]. See Table 1 for a detailed overview of input used Presumed care consumption per AE.

**Table 1:** The nature and incidence of postoperative adverse events based on the Dutch National Quality Registry for Refractive Surgery (n = 201,878), and the presumed healthcare consumption based on expert opinion.

Adverse Event	Event rates (%)	Presumed care consumption
CME	0.49	- In-person consultation (2 months) - Diagnostic OCT scan of the macula - 3 months of NSAID eyedrops
Persistent corneal edema	0.14	- Physical consultation (3 months) - Diagnostic pachymetry of the cornea - 3 months NaCl 5% eye drops - Posterior lamellar grafting surgery in 10% of cases, plus extra consultation and 3 months of steroid eyedrops
Increased IOP	0.03	- Physical consultation (3 months) - Diagnostic OCT scan of the optic nerve - 3 months of IOP lowering eyedrops
Retinal Detachment	0.03	- Physical consultation (Emergency) - Surgery: vitrectomy
Subluxation IOL	0.02	- Physical consultation (Emergency) - Surgery: ocular lens replacement
Endophthalmitis	0.02	- Physical consultation (Emergency) - Surgery: vitreous fluid replacement - Intravitreal injection of antibiotics
Wound leakage	0.01	- Physical consultation (Emergency) - Surgery: repositioning suture
Remaining lens chunk	0.11	- Physical consultation (Emergency) - Surgery: anterior chamber irrigation/aspiration
Anterior chamber flare	0.28	- Physical consultation (Emergency) - 1 month of steroid eyedrops

Reported adverse events and their rates are based on 2024 data. Accessed via https://cataract.dhd.nl and visualized in the *NOG Infographic Results of the Dutch Cataract Registration 2024*.

CME, cystoid macular edema; IOP, intraocular pressure; IOL, intraocular lens; NSAID, non-steroidal anti-inflammatory drug; OCT, optical coherence tomography.

Costs were based on Diagnostic Registration Combination codes, which are publicly available at https://opendisdata.nza.nl from the Dutch Healthcare Authority (in Dutch: *Nederlandse Zorg autoriteit*) [18]. Unit costs were calculated in accordance with the national Guideline for Economic Evaluations in Health Care and were analyzed using the Consumer Price Index based on 2024 values in euros [19]. Medication costs were based on publicly available data provided by the National Health Care Institute (in Dutch: *Zorginstituut Nederland*; https://medicijnkosten.nl) and are shown in Table 2 [20].

**Table 2:** Costs of healthcare consumption due to adverse events following cataract surgery.

	Costs (€)^1^
Consultations	
- Physical consultation (incl. diagnostics such as OCT)	€190
- Physical consultation (excl. diagnostics)	€120
Surgery^2^	
- Posterior lamellar grafting surgery	€6,275
- Vitrectomy	€2,895
- IOL replacement	€1,420
- Anterior chamber irrigation/aspiration	€1,220
- Additional suturing	€635
Medication	
- Intra-vitreal injection	€490
- 3 months of NSAIDs eyedrops	€8.80
- 3 months NaCl 5% eye drops	€15.54
- 3 months of FML eyedrops	€2.54
- 3 months of latanoprost eyedrops	€5.40
- 1 month of dexamethasone eyedrops	€3.49

^1^Assumed care products and prices were derived from the Dutch Health Care Authority (in Dutch: *Nederlandse Zorg autoriteit*) and the National Health Care Institute (in Dutch: *Nederlands Zorginstituut*).

^2^Costs of surgery include preoperative assessments and the first 6 weeks of postoperative assessments.

NSAID, non-steroidal anti-inflammatory drug; FML, fluorometholone; OCT, optical coherence tomography.

AEs were arbitrarily categorized into two groups, namely self-limiting AEs and non-self-limiting AEs. The three-month time horizon for self-limiting AEs was chosen for the direct postoperative healthcare utilization analysis based on the CORE-RCT follow-up period. Non-self-limiting AEs were considered to have potential long-term effects beyond the three months’ time horizon (e.g., structural visual impairment due to endophthalmitis); therefore, additional societal costs such as informal care by family or friends and productivity losses were structurally included. Among all AEs, only endophthalmitis and retinal detachment were classified as non-self-limiting AEs due to the high risk of irreversible visual impairment.

### Estimating the long-term costs of adverse events

Long-term annual medical costs due to visual impairment were included for non-self-limiting AEs based on Schakel et al. (2018) [21] in which the authors investigated the economic burden of visual impairment on societal costs. We included productivity losses and informal care costs in order to estimate the impact of these AEs on long-term societal cost (see S1 Table) [22,23]. Other potential long-term costs were not included due to the absence of applicable literature (e.g., nursing care, recurrent hospitalizations, or non-medical consumption in life-years gained). Productivity losses were calculated based on the Dutch labor force as follows: percentage of productivity loss due to visual impairment [24], work week [25], percentage of working population [25], percentage of cataract patients within this population [26], and the costs of one productivity hour [19]. Informal care was calculated using EU data for the average number of hours of informal care per week provided to patients with visual impairment or blindness [27], and the hourly costs associated with informal care [19]. Informal care costs per case per year were estimated at €17,010 for both endophthalmitis and retinal detachment. The cost of productivity losses was derived from Marques et al. [24]. and was estimated at €924 per non-self-limiting AE.

### Threshold analysis

The collected probability of various AEs (Table 1) were combined with the resulting costs of these AEs (Table 2) to estimate the mean current cost of AEs per patient.

A threshold analysis is a form of scenario analysis used in health economics [28] to evaluate the impact of a range of alternative values of one or two parameters on the outcome. A threshold value thereby shows under which assumptions the outcome would still hold. In this light, a threshold analysis directly informs decision makers and guideline developers regarding the robustness of their recommendations [29].

We performed several threshold analyses in which one key parameter—the incidence of AEs with remote self-assessment—was varied in each analysis. We analyzed the impact on costs when 5%-200% (double as much) of AEs were detected by an in-person postoperative assessment compared to a remote self-assessment. This impact was then combined with a range of values in the following three parameters: *i*) the hypothetical costs of remote monitoring (assumed to be €0–30 per test); *ii*) the percentage of in-person consultations needed after remote self-assessment (ranging 20%-100%; and *iii*) the underestimation of societal costs (ranging 100%-500%).

A base-case scenario was assessed in a scenario comprised of 50% avoided AEs, the cost of self-assessment of €20 per patient, 20% of patients requiring a subsequent in-person consultation, and an underestimation of societal costs of 250%.

Given that endophthalmitis typically presents as an acute emergency prior to the 4–6 week postoperative visit and retinal detachment months later in the low-risk cataract populations, both complications are expected to be identified equally across follow-up modalities, irrespective of whether care is delivered remotely or in-person) [30,31]. Therefore, we performed a sensitivity analysis restricted to AEs which are more plausible to be detected at the routine 4–6 week visit: CME, persistent corneal edema, increased IOP, IOL subluxation, wound leakage, remaining lens fragment, and anterior chamber flare.

For transparency and reproducibility, a worked example detailing the cost calculations for endophthalmitis and the base-case scenario threshold analysis is provided in S2 Table.

## Results

The cost data collected during the CORE-RCT are provided in Table 3. The difference in transportation-related costs and costs related to guidance was minimal between groups, as the patients in the self-assessment group were still requested to visit the clinic for an in-person visit, commensurate with applicable national guidelines regarding cataract care. Nevertheless, the potential differences between groups were modelled and are reported in the threshold analysis.

**Table 3:** Healthcare utilization in the CORE-RCT study.

Characteristic	Usual care, n = 63	Telemonitoring, n = 47
Transportation to the in-person follow-up visit, n (%)^1^		
*Personal vehicle*	40 (73)	31 (66)
*Public transportation*	9 (16)	9 (19)
*Taxi*	1 (1.8)	2 (4.3)
*Other*	5 (9.1)	5 (11)
Paid for parking, n (%)	26 (47)	20 (43)
Parking fee (if applicable) in €, mean (SD)	3.7 (1.3)	3.4 (1.3)
Travel distance (each direction) in km, mean (SD)	16.2 (24.4)	15.1 (15.8)
Patient guidance by a relative, n (%)^1^		
*Guidance needed*	31 (56)	27 (57)
*No guidance needed*	24 (44)	20 (43)
Guidance cancelled work (if appliable), n (%)	4 (13)	2 (7.4)
Number of postoperative visits	75	7
Reasons for unscheduled postoperative visits	Usual care (n = 12)	Telemonitoring (n = 7)
*No pathology*	3 (25)	4 (57)
*Dry eye syndrome or blepharitis*	5 (42)	1 (14)
*Postoperative inflammation*	2 (17)	1 (14)
*Intraoperative complication*	2 (17)	1 (14)

^1^Data were missing for 8 patients in the usual care group.

### Threshold analysis

The costs associated with an in-person follow-up consultation were presumed to be €120, assuming no additional diagnostic tests are indicated [19]. On average, AEs contributed an additional €16.35 to the cost per patient, with the majority due to the rare but severe complications of endophthalmitis and retinal detachment (Table 4). The base-case scenario resulted in savings in all three threshold scenarios ranging from -€83 to -€92 per patient.

**Table 4:** Costs and occurrences of postoperative adverse events following cataract surgery.

Adverse event	Cost per adverse event, €	Incidence, %	Mean cost per patient, €
Endophthalmitis	€22,238	0.02	€4.45
Retinal detachment	€23,423	0.03	€7.03
CME	€216	0.49	€1.06
Persistent corneal edema	€915	0.14	€1.28
Increased IOP	€206	0.03	€0.06
Subluxation IOL	€1,622	0.02	€0.32
Wound leakage	€508	0.01	€0.05
Remaining lens fragment	€1,410	0.11	€1.55
Anterior chamber flare	€193	0.28	€0.54
**Total**	**€50,732**	**1.13**	**€16.35**

CME, cystoid macular edema; IOP, intraocular pressure; IOL, intraocular lens.

Table 5 summarizes the outcomes of the three threshold analyses. Table 5a summarizes the analysis in which we compared the savings from not having an in-person consultation and the added costs of self-assessment versus the additional cost of potentially missed AEs that could have been prevented by having an in-person visit. Replacing the in-person consultation with a self-assessment results in cost savings in all combinations of parameter values in all threshold analyses in which we applied various self-assessment costs. Table 5b summarizes the analysis for patients who still require an in-person consultation after completing the self-assessment compared to the cost of additional missed AEs that could have been prevented. Self-assessment results in a cost savings when <80% of patients still require an in-person consultation after self-assessment, independent of the rate of missed AEs. In contrast, self-assessment results in an increase in costs only if 90–100% of patients still require an in-person consultation. Finally, Table 5c shows that even if the societal costs were underestimated, self-assessment would still result in cost savings in all combinations of parameter values.

**Table 5:** Threshold analyses per patient on self-assessments, patients in need of in-person follow-up despite self-assessment, and the underestimation of societal costs.

	Unexpected AEs avoided by an in-person consultation^1^					
**a.** Threshold analysis on the cost of self-assessment						
**Cost of self-assessment per visit**	**5%**	**25%**	**50%**	**75%**	**100%**	**200%**
€30	-€89	-€86	-€82	-€78	-€74	-€57
€20	-€99	-€96	-€92*	-€88	-€84	-€67
€10	-€109	-€106	-€102	-€98	-€94	-€77
€5	-€114	-€111	-€107	-€103	-€99	-€82
€0	-€119	-€116	-€112	-€108	-€104	-€87
**b**. Threshold analysis on patients that require an in-person consultation after performing a self-assessment						
**In-person follow-up visit needed after self-assessment**	**5%**	**25%**	**50%**	**75%**	**100%**	**200%**
20%	-€95	-€92	-€88*	-€84	-€80	-€63
40%	-€71	-€68	-€64	-€60	-€56	-€39
60%	-€47	-€44	-€40	-€36	-€32	-€15
80%	-€23	-€20	-€16	-€12	-€8	€9
100%	€1	€4	€8	€12	€16	€33
**c**. Threshold analysis on the underestimation of societal costs						
**Underestimation of societal costs**	**5%**	**25%**	**50%**	**75%**	**100%**	**200%**
100%	-€108	-€104	€100	-€96	-€92	-€76
250%	-€91	-€87	-€83*	-€79	-€75	-€59
500%	-€62	-€58	-€54	-€50	-€46	-€30

Green = a saving per patient of>€20 in the favor of remote self-assessment; yellow = a savings of €0–20; red = additional costs. In these analyses, in-person consultation costs €120 per visit.

* Combination of factors that constitute a base-case scenario.

AEs, adverse events.

^1^All AEs listed by Table 1 were used for this analysis.

When the threshold analysis was restricted to AEs plausibly detectable at the routine 4–6 week visit — excluding endophthalmitis and retinal detachment, which present independently of the follow-up modality — the mean relevant AE cost per patient was €4.87, compared to €16.35 in the primary analysis. Under these conditions, the base-case net savings increased to €94–€98 per patient (Table 6).

**Table 6:** Sensitivity analysis on the impact of excluding retinal detachments and endophthalmitis as AEs, restricting the analysis to more typically detected AEs at the routine 4–6 week postoperative visit [1].

	Base case scenario’s of Table 5	Base case scenario’s excluding endophthalmitis and Retinal Detachment^1^
a. Cost of self-assessment per visit	-€92	-€98
b. In-person follow-up visit needed after self-assessment	-€88	-€94
c. Underestimation of societal costs	-€83	N/A^2^
**Range**	-€83 up to -€92	-€94 up to -€98

Green = a saving per patient in the favor of remote self-assessments. AE = Adverse Event.

^1^Included AEs are: Cystoid Macular Edema, persistent corneal edema, increased IOP, IOL subluxation, wound leakage, remaining lens fragment, and anterior chamber flare.

^2^Not applicable as societal costs are related to long-term complications, which were endophthalmitis and retinal detachments.

## Discussion

Here, we examined the potential savings when patients perform a self-assessment following cataract surgery compared to a routine in-person 4–6-week follow-up visit by assessing the conditions in which the costs due to a potential increase in AEs due to remote follow-up would outweigh the savings realized by avoiding an in-person follow-up visit. The absolute costs associated with non-self-limiting AEs (e.g., endophthalmitis or retinal detachment) were specifically high, exceeding €21,000 per event, largely due to societal costs. Our threshold analyses suggests that while the absolute cost of a major complication is substantial when considered on a case-to-case basis, the relatively low incidence of such AEs results in a modest average of only €16 per patient, while the direct costs of routine in-person follow-up consultations are considerably higher (€120). Importantly, these findings do not suggest that in-person postoperative monitoring can or should be omitted entirely; rather, they highlight the notion that collecting essential clinical data such as patient-reported outcomes, visual acuity, and refractive status through validated self-assessment tools may provide a more efficient alternative. Based on our results, a universal in-person approach to follow-up care is not the optimal costing strategy under a wide range of assumptions regarding missed complications, telemedicine costs, and societal costs. The analyses provide insights regarding the investments needed for in-person follow-up visits, and how effective these visits are in preventing avoidable AEs. While objective measurements remain essential for postoperative quality control, our findings invite further reflection on whether postoperative cataract follow-up may eventually evolve into a more risk-stratified, hybrid model in which essential clinical outcomes are still monitored, but the mode of data collection is optimized in order to strike a balance between safety, effectiveness, and resource use.

The sensitivity analysis restricted to AEs plausibly detectable at the 4–6 week postoperative visit yielded higher estimated savings (€94–€98 per patient) compared to the primary analysis (€83–€92 per patient), reflecting the fact that the primary threshold analysis was intentionally conservative by attributing the full societal burden of endophthalmitis and retinal detachment to the follow-up modality decision. In clinical practice, however, both complications present on a timescale largely independent of the routine postoperative visit — endophthalmitis as an acute emergency and retinal detachment typically months after surgery [30,31] — and are therefore equally likely to be identified regardless of whether follow-up is delivered remotely or in-person. This distinction is clinically important, as it implies that the theoretical risk of delayed detection of these severe complications should not be considered a primary argument against implementing remote self-assessment in the low-risk cataract population.

Our analysis also indicates that societal costs—particularly costs related to informal care and productivity losses —account for the majority of costs associated with severe complications. Limited data were available to estimate these societal costs; however, an increase in societal costs would not affect our conclusions. We assessed informal care and productivity losses as from an adapted societal perspective, acknowledging that this concept is broader than these two types of costs [23]. Examples of costs that were not assessed include the potential effects of formal nursing and recurrent hospitalizations (e.g., due to falling or delirium); however, these variables are lacking in the literature [22,32].

From a clinical and systems perspective, a one-size-fits-all approach to postoperative cataract care may not be optimal. In contrast, a hybrid strategy—in which most patients are monitored remotely, with in-person follow-up care reserved for those who have a higher risk of developing complications and/or have concerning symptoms—may provide a more sustainable balance between safety, efficiency, and accessibility [9–12]. Previous research has shown that telemedicine can be a viable alternative for routine postoperative follow-up care, reducing the burden on healthcare professionals while maintaining the standards of care [8,33]. Our study builds on this notion by providing insights into the potential cost of missing complications that could have been prevented compared to the savings realized by reducing in-person care. These results are aligned with the aim of integrating telemonitoring into established guidelines and registries, such as the current ESCRS guidelines for cataract surgery.

This study has several limitations that warrant consideration. First, although this study builds on the clinical results obtained from the CORE-RCT study, it relies on information from the published literature rather than the direct observation of healthcare resource use. Ethical constraints made it necessary to perform a scenario-based analysis, rather than withholding in-person follow-up care in patients randomized to the telemedicine arm. Second, our cost calculations may have underestimated the broader consequences of visual impairment, as we were unable to monetize intangible outcomes such as falls, effects on mental health, and reduced quality of life. In addition, the model applies long-term visual impairment costs to all long-term cases of endophthalmitis and retinal detachment. Given that a substantial proportion of patients recover useful vision, this may overestimate the true long-term societal burden. We mitigated this limitation by deriving data from a study in which 74% of participants had a visual acuity better than 20/80 Snellen and only 1.9% had a visual acuity of 20/250 Snellen or worse, considerably higher compared to the post-operative VA in endophthalmitis and retinal detachment cases [34,35]. Third, although we accounted for the costs associated with informal care and productivity losses—the largest contributors to societal costs—some societal costs could not be included due to limited data availability, for example in the case of formal long-term care [28]. Fourth, successful implementation of remote follow-up care will depend on patients’ digital literacy and the availability of a suitable infrastructure, both of which can vary among populations. Future research should prospectively assess the safety, acceptability, and real-world impact of telemonitoring, particularly in a diverse range of clinical settings [36]. Fifth, this study did not account for changes in quality of life and therefore is considered an early cost analysis study. Robust quality of life estimates specifically applicable to postoperative cataract complications were not available. A cost analysis study like this can forego of the societal and ethical implications that a formal cost-effectiveness study entails. This resulted from a paucity in evidence on quality of live estimates of cataract complications. Last, this study is subject selection bias as only participants who were interested and capable in telemedicine participated. We do not consider this a limitation since patients lacking digital skills do not reflect the intended target population for this intervention. Moreover, digital accessibility among older adults is increasing rapidly; in the Netherlands, 86% of individuals aged 65–75 years currently own a smartphone with internet access, and this proportion is expected to further increase in future generations [37]. The cataract population of older age are often excluded from telemedicine under the false assumption that telemedicine is unsuitable due to limited digital literacy [7,38,39].

### Generalizability and implementation

Although this study included centers from the Netherlands, Germany, and Austria, most cost inputs were derived from Dutch healthcare costing databases. Therefore, the absolute cost savings reported may differ across healthcare systems with different reimbursement structures; especially the costs of labor and travel varies significantly. Nevertheless, because personnel costs and outpatient visits are generally relatively expensive across European healthcare systems, we expect the overall direction of our findings to remain broadly applicable.

Implementing remote eye care along the lines of the CORE-RECT creates a risk of false-negative assessments, specifically where a postoperative complication is not identified promptly. Small increases in delayed detection of vision-threatening adverse events could have clinical and economic consequences, necessitating future prospective studies that carefully address its impact in a real-world setting.

Importantly, achieving cost savings using a hybrid approach will depend on certain conditions. One such is that telemedicine should reduce the workload for personnel; however, evidence suggests that telemedicine may—in some cases—unexpectedly increase this workload [40]. Moreover, it is essential that telemedicine is safe. Nevertheless, the low rate of non-self-limiting complications indicates that a future prospective RCT is ethical and will be necessary to evaluate safety.

Our findings contribute to the larger discussion where global healthcare systems face increasing pressure to optimize their resources. With respect to cataract surgery, the ability to conduct at least part of the postoperative follow-up care remotely could be particularly beneficial in regions with limited or constrained access to care. Furthermore, integrating remote self-assessment into routine practice may serve as a steppingstone to broader applications such as AI-assisted diagnostics and automated patient triage systems.

In conclusion, our findings provide new insights supporting the notion that replacing routine in-person 4–6-week postoperative visits with a self-administered remote visit can potentially result in cost savings under a wide range of assumptions. Although prospective validation in a clinical setting is needed to confirm safety and the feasibility of implementing such a strategy, these findings support a shift toward including telemonitoring as part of a modernized approach to appropriate postoperative care in patients following cataract surgery.

## Supporting information

S1 Table Variables for assessing societal costs. (DOCX)

S2 Table Example cost calculations for the case of endophthalmitis and base-case threshold analyses. (DOCX)

S1 Data Cost calculations used for calculating cataract complications. (XLSX)

S1 Checklist CHEERS Checklist. (DOCX)
