# Supplementary material for: An economic analysis of postoperative cataract consultation using remote self-assessment
Source: PLOS Digit Health. 2026 Aug 14;5(8):e0001617. doi: 10.1371/journal.pdig.0001617 (PMC13475914; doi:10.1371/journal.pdig.0001617)
Supplement: Supplementary file 1 [file pdig.0001617.s001.docx]

| **Supplementary** **Table S1. Variables for assessing societal costs** | | |
| --- | --- | --- |
| **Variables** | **Values** | **Source** |
| Time spent at informal care, hours per week (range)^1^ | 17,4 (5,8-94,1) | (Schmier et al. 2006) |
| Informal care hour rate, € | 18,80 | (Zorginstituut Nederland 2024) |
| Productivity hour rate, € | 39,88 | (Zorginstituut Nederland 2024) |
| Productivity loss due to VA decrease, % | 20,58 | (Marques et al. 2021) |
| Dutch population in employed labour force, % | 70,40 | (Borger et al. 2003) |
| Cataract population in labour force, % | 10,60 | (Borger et al. 2003) |
| Work week, hours per week | 32,10 | (CBS StatLine 2024) |
| Work year, weeks per year | 47 | (Zorginstituut Nederland 2024) |
| Yearly long-term medical costs due to VI^2^ | 523 | (Schakel et al. 2018) |
| ^1^ Average hours help received per week of n=803 respondents, of which 74% had a VA better than 20/80 Snellen, and 1,9% of respondents had a VA of >250 Snellen or worse.  ^2^ Applicable for non-self-limiting adverse events | | |
