# Supplementary material for: An economic analysis of postoperative cataract consultation using remote self-assessment
Source: PLOS Digit Health. 2026 Aug 14;5(8):e0001617. doi: 10.1371/journal.pdig.0001617 (PMC13475914; doi:10.1371/journal.pdig.0001617)
Supplement: Supplementary file 2 [file pdig.0001617.s002.docx]

| **Cost Components for the case of endophthalmitis** | **Source** | **Amount (€)** |
| --- | --- | --- |
| Surgery | Table 1 & 2 | €1,220 |
| Intravitreal injection |  | €490 |
| Outpatient consultation |  | €190 |
| **Subtotal: Healthcare costs** |  | **€1,900** |
| Informal care costs | 17.4 hrs/wk × €18.80 × 52 wks (Suppl. Table S1) | €17,010 |
| Societal productivity costs | 10% of cataract population in working age (Suppl. Table S1) | €924 |
| Annual medical costs (visual impairment) | Schakel et al., 2018 (Suppl. Table S1) | €2,404 |
| **Total costs per complication** |  | **€22,238** |
| Incidence | Table 1 | 0.02% |
| Mean cost per patient | Table 4 | €4.45 |
| **Total mean costs per patient (all complications combined)** | Table 4 | **€16.35** |
| **Example calculations Threshold analyses** |  | **Net saving (€)** |
| **5a) Costs of self-assessment per visit**  Consultation - self-assessment cost; 50% reduction in adverse events | €120 − €20 − (€16.35 × 50%) | €92 |
| **5b) In-person follow-up visit needed after self-assessment**  20% of patients require additional in-person consultation; 50% reduction in adverse events | €120 − (€120 × 20%) − (€16.35 × 50%) | €88 |
| **5c) Underestimation of societal costs**  Societal costs of and retinal detachment underestimated by 250%; 50% reduction in adverse events | €120 − [(€4.45 + €7.03) × 250%] × 50% − (€16.35 × 50%) | €83 |
| **Supplementary Table S2. Example cost calculations for the case of endophthalmitis and base-case threshold analyses.** | | |
